# Environmental Hazards of Nanobiomaterials (Hydroxyapatite-Based NMs)—A Case Study with *Folsomia candida*—Effects from Long Term Exposure

**DOI:** 10.3390/toxics10110704

**Published:** 2022-11-18

**Authors:** Bruno Guimarães, Susana I. L. Gomes, Elisabetta Campodoni, Monica Sandri, Simone Sprio, Magda Blosi, Anna L. Costa, Mónica J. B. Amorim, Janeck J. Scott-Fordsmand

**Affiliations:** 1 Department of Biology & CESAM, University of Aveiro, 3810-193 Aveiro, Portugal; 2 National Research Council, Institute of Science and Technology for Ceramics, 48018 Faenza, RA, Italy; 3 Department of Ecoscience, Aarhus University, C.F. Møllers Alle, DK-8000 Aarhus, Denmark

**Keywords:** ecotoxicology, soil, invertebrates, long-term toxicity, nanobiomaterials

## Abstract

Hydroxyapatite (HA) is a calcium phosphate used in many fields, including biomedical applications. In particular, ion-doped HA nanomaterials (nHA) are developed for their increased bioactivity, particularly in the fields of regenerative medicine and nanomedicine. In this study, we assessed the ecotoxicological impact of five nHA materials: a synthesized calcium hydroxyapatite (CaP-HA), superparamagnetic iron-doped hydroxyapatite (Fe-HA), titanium-doped hydroxyapatite (Ti-HA), alginate/titanium-doped hydroxyapatite hybrid composite (Ti-HA-Alg), and a commercial HA. The soil ecotoxicology model species *Folsomia candida* (Collembola) was used, and besides the standard reproduction test (28 days), an extension to the standard for one more generation was performed (56 days). Assessed endpoints included the standard survival and reproduction, and additionally, growth. Exposure via the standard (28 days) did not cause toxicity, but reproduction increased in commercial HA (significantly at 320 mg HA/kg) whereas via the extension (56 days) it decreased in all tested concentrations. Juveniles’ size (56 days) was reduced in all tested nHA materials, except commercial HA. nHA materials seem to trigger a compromise between reproduction and growth. Long-term effects could not be predicted based on the standard shorter exposure; hence, the testing of at least two generations (56 days) is recommended to assess the toxicity of nanomaterials, particularly in *F. candida*. Further, we found that the inclusion of size as additional endpoint is highly relevant.

## 1. Introduction

Nanotechnology has also been revolutionizing the biomedical industry, through the development of nanobiomaterials (NBMs)—natural or synthetic nanoscale materials that are highly biocompatible [1]. The list of potential applications for NBMs is vast and includes, for instance, tissue engineering, cancer therapy, and also therapeutics for novel viruses, such as SARS-CoV2 [1].

Hydroxyapatite (HA) is a calcium phosphate compound (Ca_10_(PO_4_)_6_(OH)_2_) used in many fields, including biomedicine [2]. Due to its biocompatibility and chemical similarity to the mineral component of human bone and teeth, HA is used in orthopaedic, dental and maxillofacial applications [2]. Many studies reported biomedical applications of HA as a bone repairing material [3,4,5,6] or biocompatible coating for bioimplant materials, due to its ability to provide support for bone in-growth and osteointegration [7,8]. HA can be produced using advanced technologies and low-cost raw materials, including fish bones and industrial by-products, hence making HA economically viable for application in the remediation of contaminated fields [9]. HA has also several applications in cosmetics, as a booster of the sun protection factor (SPF) [10], and in pharmaceutical industries where it is used as a protein delivery media [11,12,13] and drug releasing agent [14,15,16]. Additionally, other biomedical applications emerge from HA composite materials, e.g., TiO_2_-HA were developed to improve the bonding of implant materials [17].

HA nanoparticles/nanomaterials (nHA) present several advantages, such as the superior surface area, high stability under oxidizing and reducing conditions, and excellent cytocompatibility, with one main disadvantage being its tendency for agglomeration [18,19]. Ion doping of nHA can enhance biologic and functional properties, e.g., Fe-doped nHA has a more negative surface charge which increases its adhesion in osteoblastic cells in comparison to pure nHA; in addition, Fe ions, when present in both oxidation states (II, III), can induce superparamagnetic behaviour in nHA and further stimulate the differentiation and proliferation of stem cells [20,21,22].

Despite the recognised advantages of using nHA, there are environmental concerns. For instance, nHA were demonstrated to delay hatching in zebrafish (*Danio rerio*) embryos [23], and to distress the neuronal development and behaviour of the fruit fly *Drosophila melanogaster* [24]. nHA, as other nanomaterials, can reach the soil by direct spill (including accidental) during production, use and release of NMs and NMs containing products, or also indirectly by emission via the effluent of waste water treatment plants (WWTPs), application of biosolids to soil, or leachates from landfills [25]. After reaching the soil, nHA is not expected to be leached down more than 5% [26], hence the animals inhabiting the top layer of the soil are at high risk. Studies on the toxic effects of nHA to soil living invertebrates are currently absent. Further, there has been a growing concern on the long-term effects of stressors, particularly chemicals, and the importance of multigenerational exposure is well recognized [27].

In the present study, we aimed to assess the effects of five nHA materials to the standard model species *Folsomia candida* (Collembola), based on the standard 28 days reproduction test [28] and the 56 days of standard extension [29,30]. *F. candida* has a worldwide distribution and is a model species in the soil ecotoxicology studies for more than 40 years. Because it is a detritivore arthropod that lives throughout the upper soil profile, it was selected due to being a non-target species that is a potential receptor for nHA. The assessed endpoints included survival, reproduction and size. The tested nHA include: (1) a commercial hydroxyapatite (Sigma-HA), (2) an undoped calcium hydroxyapatite obtained by wet synthesis (CaP-HA) plus three ion-doped nHA materials, i.e., (3) superparamagnetic iron-doped hydroxyapatite (Fe-HA), (4) titanium-doped hydroxyapatite (Ti-HA), and (5) an alginate/titanium-doped hydroxyapatite hybrid composite (Ti-HA-Alg). We here hypothesize that the toxicity of the different nHA materials will increase after long-term exposure.

## 2. Materials and Methods

### 2.1. Test Organisms

The standard test species *Folsomia candida* (Collembola) was used. Organisms were cultured on a moist substrate of the plaster of Paris and activated charcoal (8:1 ratio), at 20 ± 1 °C, under a photoperiod of 16:8 h (light:dark). Individuals were fed weekly with dried baker’s yeast (*Saccharomyces cerevisiae*). Cultures were synchronized to obtain age synchronized juveniles (10–12 days old).

### 2.2. Test Soil

The natural standard LUFA 2.2 soil (LUFA Speyer, Germany) was used for the experiments and is characterized as follows: pH (0.01 M CaCl_2_): 5.6 ± 0.4; organic carbon: 1.71 ± 0.30%; cation exchange capacity (CEC): 9.2 ± 1.4 meq/100 g; maximum water holding capacity (maxWHC): 44.8 ± 2.9 g/100 g; texture: 8.0 ± 1.5% clay, 13.7 ± 1.0% silt, and 78.3 ± 1.0% sand content.

### 2.3. Test Materials, Synthesis and Characterization

The tested materials (Table 1) included the commercial nanomaterial hydroxyapatite (Sigma-HA) (Sigma-Aldrich, Merck Life Science S.L.U., Portugal, ≥97% synthetic, nanopowder, ≤200 nm particle size (BET), ≥9.4 m^2^/g surface area) as a reference and the lab scale prepared materials: calcium hydroxyapatite (CaP-HA), superparamagnetic iron-doped hydroxyapatite (Fe-HA), titanium-doped hydroxyapatite (Ti-HA) and alginate/titanium-doped hydroxyapatite hybrid composite (Ti-HA-Alg), all powders (for full details please see Supplementary Materials).

CaP-HA was prepared by a previously described neutralization process carried out at 40 °C [31], involving calcium hydroxide (Ca(OH)_2_, 95 wt.%, Sigma-Aldrich, Saint Louis, MO, USA) and phosphoric acid (H_3_PO_4_, 85 wt.%, Sigma Aldrich, Saint Louis, MO, USA) aqueous solutions, and prepared to have Ca/P molar ratio = 1.67. The acid solution was dropped into the alkaline suspension under stirring. After neutralization, the powder suspension was left under stirring for 3 h in the mother solution and then aged for 24 h. Then, the solid was washed with deionized water, then the solid was dried and sieved at 150 μm.

Fe-HA was prepared by a similar neutralization method, by also adding to the alkaline Ca(OH)_2_ suspension an aqueous solutions containing FeCl_2_·4H_2_O (99 wt.%, Sigma Aldrich, Saint Louis, MO, USA) and FeCl_3_·6H_2_O (97 wt.%, Sigma Aldrich, Saint Louis, MO, USA) as a source of Fe^2+^/Fe^3+^ ions, and prepared to have molar Fe/Ca ratio = 0.2 [32]; after neutralization was obtained by dripping the H_3_PO_4_ solution under continuous stirring, the powder suspension was washed with water, then the solid was dried and sieved at 150 μm.

Ti-HA was prepared by a similar neutralization method, by also adding a titanium isopropoxide solution (97 wt.%, Alfa Aesar GmbH & Co KG, Germany) dissolved in isopropyl alcohol (97 wt.%, Sigma-Aldrich, Saint Louis, MO, USA) and dripped into the alkaline solution kept at 45 °C, as previously described [33].

Ti-HA-Alg was obtained by inducing heterogeneous nucleation of Ti-HA on sodium alginate (alginic acid sodium salt from brown algae; Sigma Aldrich, Saint Louis, MO, USA); briefly, the alginate solution (4 g in 100 mL of H_2_O) was prepared under magnetic stirring and ultrasound bath. A titanium solution was prepared quickly and under Argon flux to avoid the oxidation of Ti^4+^ (2.67 g in 12.7 mL of isopropanol (C_3_H_8_O, Sigma Aldrich, Saint Louis, MO, USA). Then, the phosphoric acid solution (4.15 g in 30 mL of H_2_O) and the titanium solution were dripped simultaneously into the Ca(OH)_2_ suspension (4.723 g in 100 mL of H_2_O), kept at 45 °C. Then, the alginate solution was also added dropwise to the basic suspension. The product was left stirring for 2 h, and then left ageing without stirring and heating for 2 h. After 3 cycles of washing with deionized water, the Ti-HA-Alg product was freeze-dried by setting the cooling temperature at −40 °C and the heating ramp (2 °C min^−1^ up to −5 and 1 °C min^−1^ up to 25 °C) at *p* = 0.086 mbar to achieve the final Ti-HA-Alg hybrid flakes. For further details, please see Supplementary Materials.

### 2.4. Spiking Procedure

Spiking followed the recommendations for nanomaterials [34], i.e., materials (dry powders) were mixed with dry soil, and per replicate, i.e., prepared individually (to ensure total raw amounts of the tested material). In short, 30 g of soil per replicate was thoroughly mixed with the corresponding amount of the test materials (as dry powders) to obtain the final concentration range: 0–100–320–1000–3200 mg/kg for all nHA tested materials. Deionised water was added to achieve 50% of soil maxWHC and the soil was homogeneously mixed. Soil was left to equilibrate for 1-day prior to the test start.

### 2.5. Test Procedure

Tests were conducted following the standard OECD guideline 232 [28] plus an extension, as described in Guimarães et al. [29,30], representing one more generation compared to the standard. In short, each test vessel contained 30 g of moist soil with food (baker’s yeast) and 10 juveniles were introduced; the vessel was covered with parafilm with holes to allow aeration. Four replicates per treatment were conducted for sampling day 28 (standard) and 56 (extended) and 1 replicate per monitoring on day 7, 14 and 21. For the 56-day sampling, juveniles were sampled at day 28 and further exposed for 28 more days (in soil previously spiked at day −1). The endpoints assessed were survival and reproduction. Additionally, growth (size, area, mm^2^) was measured in days 28 and 56 samples for adults and juveniles. Exposure ran at a 20 ± 2 °C and 16:8 h (light:dark) photoperiod. Food and water loss were replenished weekly. On the sampling days, test vessels were flooded with water and the content was transferred to a crystallizer dish. The surface was photographed for further automatic analyses (count and measure) using the software ImageJ [35]. At day 28, after photographing, all juveniles were collected with a spoon and transferred to a box with a layer of Plaster of Paris (culture medium), to adsorb extra water from the spoon. Animals were exposed for one more generation, selecting ten of the biggest juveniles (ca. 11 days old) and transferring them to new test vessels, where the test ran for 28 days under the same conditions.

### 2.6. Data Analysis

One-way analysis of variance (ANOVA), followed by the Dunnets’ Post-Hoc test was used to assess differences between control and treatments for each endpoint. To assess differences between 28 and 56 days of exposure, an ANOVA followed by the Holm–Sidak post hoc test was performed [36].

## 3. Results

### 3.1. Materials’ Characterization

Among the tested materials, the commercial Sigma-HA forms the largest agglomerates (hydrodynamic diameter of 2400 nm), associated with a more neutral (close to zero) surface charge. Conversely, CaP-HA, Fe-HA and Ti-HA are negatively charged and form smaller agglomerates in suspension (hydrodynamic diameters ranging from 160 to 320 nm). A summary of the main characteristics of tested materials is presented in Table 1; for the full details of characterization, please see the Supplementary Information (and Figures S1–S5).

### 3.2. Toxicity Tests

Validity criteria were fulfilled according to the guideline for the standard tests (mortality < 20%, number of juveniles > 100, and coefficient of variation < 30%). The pH did not vary significantly during the tests.

**Figure 1:**
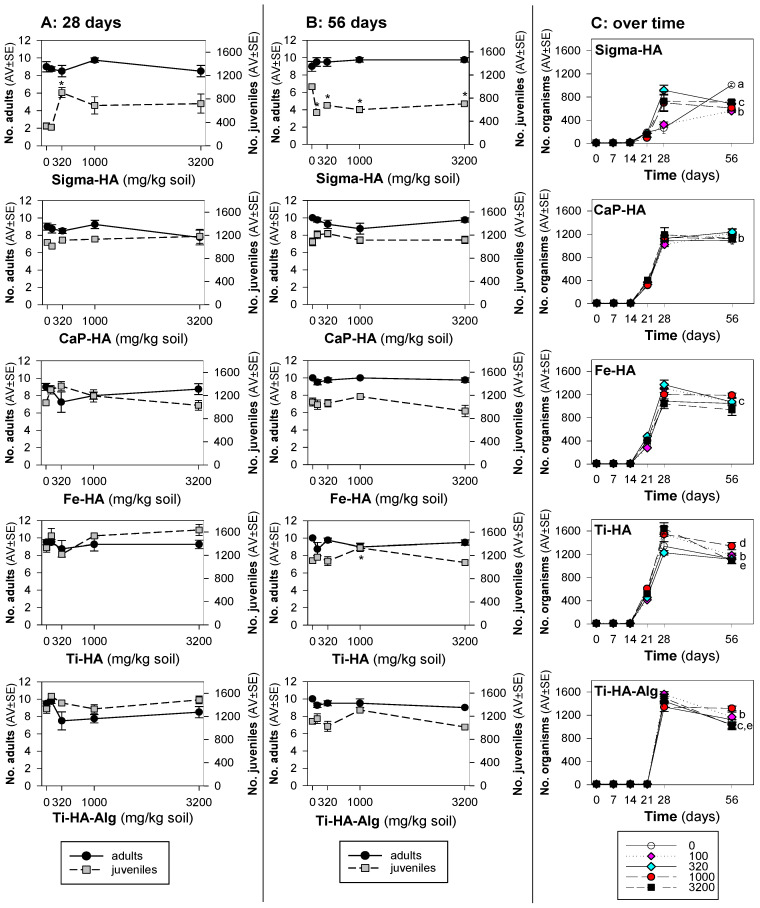
Results of the reproduction test with *Folsomia candida* when exposed in LUFA 2.2 soil to the commercial hydroxyapatite (Sigma-HA), calcium hydroxyapatite (CaP-HA), superparamagnetic iron-doped hydroxyapatite (Fe-HA), titanium-doped hydroxyapatite (Ti-HA), and alginate/titanium-doped hydroxyapatite hybrid composite (Ti-HA-Alg) in terms of number of adults and juveniles after (**A**) 28 days (standard), (**B**) 56 days (extension), and in terms of total number of organisms at (**C**) 0, 7, 14, 21, 28 and 56 days of exposure. Values are expressed as average ± standard error (AV ± SE). * (*p* < 0.05, Dunnets’): between treatments and control; a, b, c, d, e (*p* < 0.05, Holm-Sidak’): between days 28 and 56 for 0, 100, 320, 1000 and 3200 mg/kg, respectively.

For the standard (28 days) exposure, the tested materials caused little to no effect on survival or reproduction, except for Sigma-HA, where there was an increase in reproduction at 320 mg/kg and onwards (Figure 1A).

On the other hand, for the standard extended (56 days) exposure (Figure 1B), Sigma-HA caused a significant decrease in reproduction from the lowest tested concentration (100 mg/kg), and then a similar reduction throughout all tested concentrations (up to 3200 mg/kg).

The overview from the total number of organisms–population–over time (Figure 1C) allows one to see variations of pattern between day 28 and 56, but the most pronounced was for Sigma-HA, as described.

**Figure 2:**
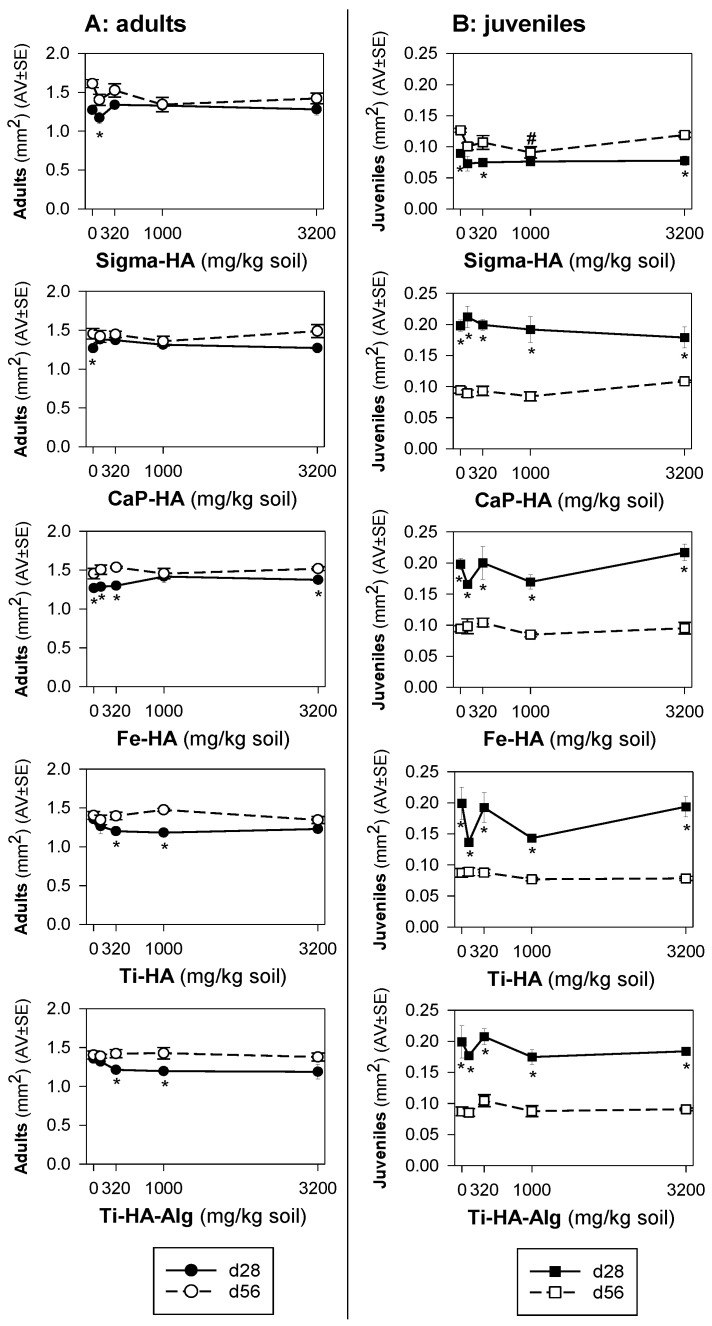
Results of the reproduction test with *Folsomia candida* when exposed in LUFA 2.2 soil to commercial hydroxyapatite (Sigma-HA), calcium hydroxyapatite (CaP-HA), superparamagnetic iron-doped hydroxyapatite (Fe-HA), titanium-doped hydroxyapatite (Ti-HA), and alginate/titanium-doped hydroxyapatite hybrid composite (Ti-HA-Alg), for 28 (standard) and 56 days (extension), in terms of size (area) of (**A**) adults; (**B**) juveniles. Values are expressed as average ± standard error (AV ± SE). * (*p* < 0.05, Holm–Sidak test): between days 28 and 56, # (*p* < 0.05, Dunnets’ test): between treatments and control.

In terms of size (Figure 2), the overall average size of adults was larger at day 56 compared to day 28 in all tested nHA materials. For juveniles, the opposite overall result was observed, i.e., in average smaller animals, at day 56, except for Sigma-HA.

On a more detailed inspection at the dose level, in the 28-day (but not at the 56-day) exposure, lower survival and negative impact on growth occurred simultaneously.

Of interest to note is that in the 56-day exposure, for Sigma-HA, where there was a decrease in the number of juveniles (reproduction, day 56), the size of the animals was similar (although slightly bigger); on the other hand, all other nHA materials did not affect the number of juveniles, but these were significantly smaller than at day 28.

## 4. Discussion

The prolonged exposure (for one additional generation compared to the standard: 56 instead of 28 days) demonstrated toxicity to certain nHA materials. Overall, Sigma-HA caused the most distinct toxicity pattern, while the other tested nHA forms induced similar effects. There was a reduced reproduction for Sigma-HA, from 100 mg/kg. On the other hand, for the 28 days of exposure, Sigma-HA induced an increase in reproduction (significant at 320 mg HA/kg), but with an impact on size, i.e., smaller juveniles. This type of R strategy, in which animals allocate energy for reproduction in detriment of growth, has been reported before. For example, a study with *F. candida* exposed to ivermectin for two generations [29] demonstrated that at 1 mg/kg there was an increase in the number of juveniles, but with a reduced size. The reason for this higher toxicity of Sigma-HA is not clear. HA materials are known to have a relatively low surface area and limited adsorption capacity [37], are not mobile in soil and reduce organic matter, which causes an imbalance in the nutrients/elements in soil [38]. If the impact of a reduction of organic matter in soil was to occur to a higher extent for Sigma-HA compared to the other doped-nHA materials, the same would have been expected for CaP-HA, i.e., the undoped analogue of nHAs, which was not the case.

Moreover, of importance to note is the large decrease in the size of the juveniles from 28 to 56 days for all the apatite samples, except for Sigma-HA (for which the juveniles were larger at 56 than at 28 days). This indicates a shift in energy allocation from growth to detoxification mechanisms. Further, the lesser effects at day 56, despite the size reduction at day 28 for all the nHA except Sigma-HA, could occur because in the implemented design the 10 largest juveniles were selected for the exposure in the next generation. Hence, longer-term effects cannot be ruled out. For Ti-HA and Ti-HA-Alg, the decrease in the population from day 28 to 56 suggests that Ti could be a source of toxicity.

Overall, Sigma-HA was the most toxic, particularly after long-term exposure. The DLS data showed that Sigma-HA is the nHA form with the highest potential to form larger aggregates in water, which could indicate that the smaller size was not related to higher effects (to note, all nHA were added to soil as dry forms and the real behaviour thereafter is unknown). In fact, current results indicate that the chemical signature of the nHA is probably the variable that seems to best explain the differences in toxicity—all nHA were produced based on the CaP-HA synthesis except Sigma-HA.

The few literature data on HA effects in vivo indicate short-term toxicity. For instance, *D. melanogaster* was exposed to HA through food suffered oxidative stress after 84 h, developmental delays in the late third instar larvae, and reduction of the body weight, as well as several phenotypical abnormalities [24]. In a study with needle-(nHA-ND) and rod-shaped (nHA-RD) HA NPs, Zhao et al. [23] reported development and hatching delays in zebrafish embryos at 72 h post fertilization (hpf), although without mortality, as assessed at 120 hpf. For instance, for alginate containing nHA (HA-Alg), no toxicity was reported, causing no effects after dietary exposure for 28 days in rabbits, in terms of body weight, fur, food consumption and water intake, bowel movement, morbidity, and mortality [39]. Although a direct comparison cannot be made (different test species, exposure routes, media, test design, etc.), our results indicate that for *F. candida* exposed through soil, the toxicity of nHA materials can occur after longer-term exposure periods. The overall low toxicity could be also due to low exposure if high aggregation of the materials occurs in the soil (as suggested by DLS data), and hence this is important to note.

## 5. Conclusions

The nHA composite materials presented low to no toxicity to collembolans based on the standard OECD test (28 days). However, with a 2-fold increase in exposure time (56 days), harmful effects on reproduction were observed for certain tested materials, particularly Sigma-HA. Moreover, all nHA tested materials impacted size, i.e., animals’ growth, either on the adults at the 28-day exposure or on the juveniles at the 56-day exposure, confirming ongoing shifts of allocation of resources between the number of juveniles and their size, e.g., more juveniles but smaller. Based on the results of this study, we recommend performing long-term exposure tests to assess the toxic effects of nanomaterials, particularly calcium phosphate materials such as hydroxyapatite to soil invertebrates. Further, we found that size was a sensitive endpoint and we recommend its addition to the standard OECD as an annex, besides the extension of the duration of the OECD test (at least for a second generation).

## Figures and Tables

**Table 1:** Main properties of the tested materials (Sigma-HA: commercial hydroxyapatite; CaP-HA: calcium hydroxyapatite; Fe-HA: superparamagnetic iron-doped hydroxyapatite; Ti-HA: titanium-doped hydroxyapatite; Ti-HA-Alg: alginate/titanium-doped hydroxyapatite hybrid composite). DLS: dynamic light scattering; ELS: electrophoretic light scattering; ICP-OES (^a^): inductively coupled plasma optical emission spectrometry; TGA (^b^): thermogravimetric analysis; n/a: not available.

Test Material	Composition	Size		Surface Charge (mV)
		DLS (nm)	X-ray sedimen-tography (μm)	ELS ζ potential
Sigma-HA	n/a	2441	n/a	1.35
CaP-HA	Ca = 34 ^a^, P = 16 ^a^, Ca/P =1.64 ^a^ (mol/mol)	320	d_90_ = 1.83 ^b^, d_50_ = 1.22 ^b^, d_10_ = 0.38 ^b^	−19.6
Fe-HA	Ca = 23 ^a^, P = 15 ^a^, Fe = 10 ^a^, (Ca + Fe)/P= 1.64 ^a^ (mol/mol)	164	d_90_ = 1.45, d_50_ = 1.16, d_10_ = 0.87	−19.3
Ti-HA	Ca= 37 ^a^, P = 14.6 ^a^, Ti = 4.5 ^a^, (Ca + Ti)/(P + Ti)= 1.49 mol/mol ^a^.	242	d_90_ = 1.54, d_50_ = 0.29	−9.11
Ti-HA-Alg	Ca/P = 1.60 mol ^a^; (Ca + Ti)/(P + Ti)= 1.49 mol/mol ^a^; Ti/Ca = 15 (mol%) ^a^. Ti/P = 23.2 (mol%) ^a^. HA: Alg= 90:10 ^b^	n/a	n/a	n/a

## Data Availability

The data presented in this study are available on request from the corresponding author.

## Supplementary Materials

The following supporting information can be downloaded at: https://www.mdpi.com/article/10.3390/toxics10110704/s1, Detailed characterization of the tested materials. Figure S1: Images of Sigma-HA via Transmission Electron Microscopy (TEM): (A,B) and via Scanning Electron Microscopy (SEM): (C,D); Figure S2: CaP-HA NM characterization via (A) SEM-FEG image, (B) XRD spectrum, and (C) FTIR spectrum; Figure S3: Fe-HA NM characterization via (A) SEM-FEG image, (B) XRD spectrum, and (C) FTIR spectrum; Figure S4: Ti-HA NM characterization via (A) FEG-SEM image, (B) XRD spectrum, and (C) FTIR spectrum; Figure S5: Ti-HA-Alg NM characterization via (A) FEG-SEM image, (B) TGA spectrum, (C) XRD spectrum, and (D) FTIR spectrum. Reference [40] are cited in the supplementary materials.
