# Supplementary material for: Environmental Hazards of Nanobiomaterials (Hydroxyapatite-Based NMs)—A Case Study with Folsomia candida—Effects from Long Term Exposure
Source: Toxics. 2022 Nov 18;10(11):704. doi: 10.3390/toxics10110704 (PMC9693573; doi:10.3390/toxics10110704)
Supplement: Supplementary file 1 [file toxics-10-00704-s001.zip › toxics-1982275-supplementary.pdf]

Supplementary Information

Environmental Hazards of Nanobiomaterials (Hydroxyapatite-Based NMs)—A Case Study with *Folsomia candida*—Effects from Long Term Exposure

Bruno Guimarães[1], Susana I. L. Gomes[1], Elisabetta Campodoni[2], Monica Sandri[2], Simone Sprio[2], Magda Blosi[2], Anna L. Costa[2], Mónica J. B. Amorim[1*], Janeck J. Scott-Fordsmand[3]

[1]University of Aveiro, Department of Biology & CESAM, 3810-193 Aveiro, Portugal.

[2]National Research Council, Institute of Science and Technology for Ceramics, 48018 Faenza (RA), Italy.

[3]Aarhus University, Department of Ecoscience, Aarhus University, C.F. Møllers Alle, DK-8000, Aarhus, Denmark

* corresponding author: *Email:* mjamorim@ua.pt

Materials and Methods

Test Materials

Phosphoric acid (H_3PO_4, 85 wt.%), calcium hydroxide ($Ca(OH)_2$, 95 wt.%), Titanium(IV) isopropoxide ($Ti(iOPr)_4$, 97wt%), Isopropyl alcohol ($(CH_3)_2CHOH$, 99.5 wt%), Iron(III) chloride hexahydrate ($FeCl_3 \cdot 6H_2O$, >98wt%), Iron(II) chloride tetrahydrate ($FeCl_2 \cdot 4H_2O$, > 99wt%) and Alginic acid sodium salt from brown algae were purchased from Sigma Aldrich (USA). Ultrapure water (0.22 mS, 25°C) was used in all experiments.

Materials characterization

Nanomaterials were characterized by Transmission Electron Microscopy (TEM), Scanning Electron Microscopy (SEM), Field emission-scanning electron microscopy (FE-SEM), X-ray Diffraction Spectroscopy (XRD), Dynamic Light Scattering (DLS), zeta-potential, Thermo gravimetric Analysis (TGA), and UV-Vis spectroscopy.

DLS measurements were carried out with a Zeta-Sizer Malvern Instrument in backscattering mode. All studies were performed at a 173° scattering angle with temperature controlled at 25 °C in 1 mL polystyrene cuvettes. Nanoparticles were characterized in terms of size and charge (Zeta-potential). Short time measurements were carried out for a total of 15 min, with 3 consecutive measurements for each sample. Zeta-potential measurements were performed in auto-mode at 25 °C, with 3 consecutive measurements for each sample. For TEM analysis, normal and ultra-thin plasma coated carbon film were used. The sample suspensions for DLS analysis were prepared by dispersing 100 mg of powder in 10 mL of citrate buffer solution at pH 6.2 and treating them with an ultrasound probe for 5 minutes, then the solid was separated from the solution by centrifuging, washed with Milli-Q water and centrifuged again, and finally was dispersed in 100 mL of Milli-Q water; the sample suspensions for ELS analysis were prepared by dispersing 15 mg of powder in 15 mL of Milli-Q water and treating them with an ultrasound probe for 5 minutes.

TEM was performed by using a JEOL-JEM 1010 microscope operating at an acceleration voltage of 100 kV. TEM images were acquired to measure size and characterize nanomaterial morphology. SEM images were acquired by JEOL JSM-6490LV, equipped with SE and BE detectors and an OXFORD INCA EDXS system is used for surface studies. SEM images were acquired to measure size and characterize nanomaterial morphology. FE-SEM images were performed on an instrument Carl Zeiss Sigma NTS Gmbh, coupled with scanning transmission electron microscopy (STEM) detector. TGA was performed on a Thermo Gravimetric Analyser (TGA) (STA449/C Jupiter, Netzsch, Germany). Briefly, 20mg of the composite was placed in aluminium crucible and then crushed and pressed for complete contact with the crucible. The experiment was carried in the temperature range of 30−800◦C at a heating rate of 10◦C/min in air atmosphere. FTIR spectrum was recorded using a Nicolet 380 FTIR spectrometer (Thermo Fisher Scientific Inc., Waltham, USA). Briefly, 2 mg of the composite was mixed with 100 mg of anhydrous potassium bromide and then pressed at 8000 psi into 7 mm diameter pellets. All spectra were recorded in the wavelength ranging from 400 to 4000 cm−1 with 2cm−1 of

resolution. XRD patterns were recorded by the Bruker D8 Advance diffractometer (Germany) operating in q/2q configuration, with a Lynx Eye detector (20-80° 2q range, 0.02 stepsize, 16 s time-per-step equivalent). The elemental analysis was carried out by Inductively Coupled Plasma-Optical Emission Spectrometry (ICP-OES) (Agilent 5100, Agilent Technologies, Santa Clara, CA, US). 20 mg of samples were dissolved in 2 mL of nitric acid (65% purity) and the solution volume was increased up to 100 mL with Milli-Q water. The analytical emission wavelengths were: Ca 422.673 nm, P 213.618 nm, Fe 259.940 nm (only for FeHA-NPs), and Ti 334.941 nm (only for TiHA-Nps).

Results

Sigma-HA were characterized by DLS, zeta-potential, TEM and SEM.

The hydrodynamic diameter of Sigma-HA [1 mg/L in MQ water] is 2441 nm, and the zeta-potential is close to zero, 1.35 + 0.1 mV. TEM and SEM images of Sigma-HA (Fig. S1) show that the particles are polydispersed and aggregated.

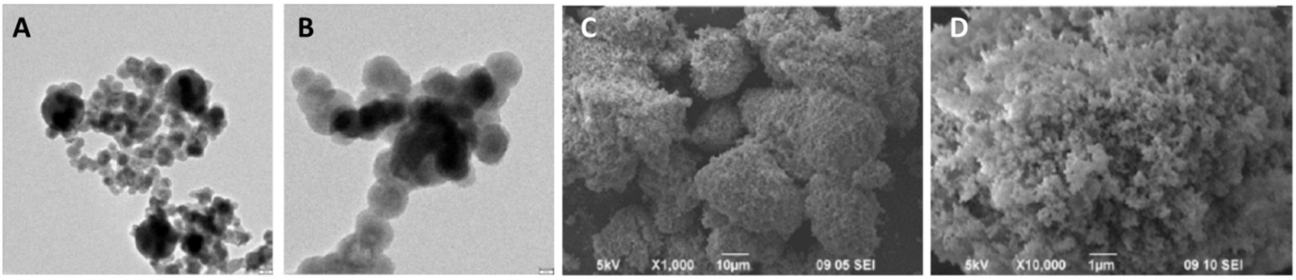

Figure S1: Images of Sigma-HA via Transmission Electron Microscopy (TEM): A) and B) and via Scanning Electron Microscopy (SEM): C and D).

CaP-HA were characterized by DLS, zeta-potential, SEM, XRD and FTIR.

CaP-HA NM were synthetized following the protocol reported by *Landi et al.* [1] appearing as a white to off-white pure crystalline powdered solid characterized by round particles with a size of 50-100 nm agglomerated onto micron-size clusters (fig. S2A).

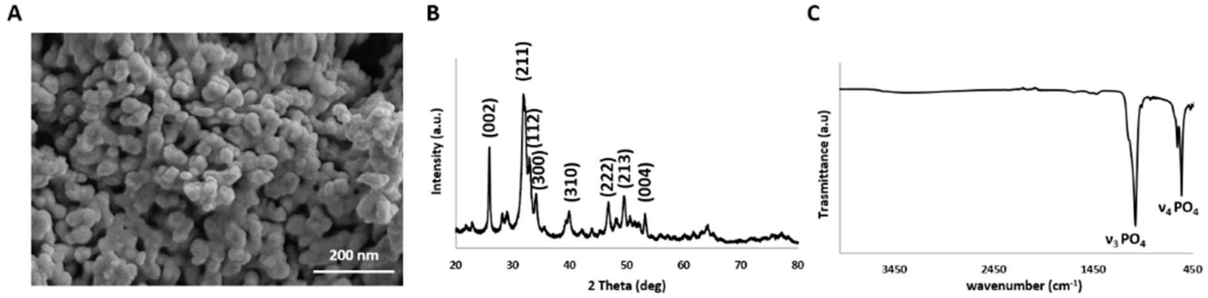

Figure S2: CaP-HA NM characterization via A) SEM-FEG image, B) XRD spectrum and C) FTIR spectrum.

The diffraction peaks are broad and poorly defined, indicating the presence of nanocrystals (about 20 nm) with reduced crystal domains and crystalline order (Fig. S2B). The hydrodynamic diameter of CaP-HA [1 mg/mL] is 319.36 nm, and the zeta-potential is -19.66 mV. The particles showed a chemical composition of: Ca = 34 ± 0.5 wt%; P = 16 ± 0.5 wt%; Ca/P molar ratio = 1.64 ± 0.02 (obtained through ICP-OES).

CaP-HA NM displayed a main broad band at 1030 cm^{-1} due to antisymmetric stretching mode of the apatitic PO_4 groups ($\nu_3 PO_4$). Other features emerge at 603 and 565 cm^{-1} (triply degenerated bending mode of the same groups, ($\nu_4 PO_4$) (Fig. S2C).

Fe-HA were characterized by DLS, zeta-potential, SEM, XRD and FTIR.

Fe-HA NM were synthetized following the protocol reported by *Tampieri et al.* [2] appearing as a brownish poorly crystalline powdered solid characterized by needle-like nanoparticles with a long axis of 75-100 nm and a short axis of 15-20 nm, agglomerated onto micron-size clusters (Fig. S3A) with the occurrence of round-shaped nanoparticles of maghemite (diameter 5 nm), clearly visible in the TEM inset.

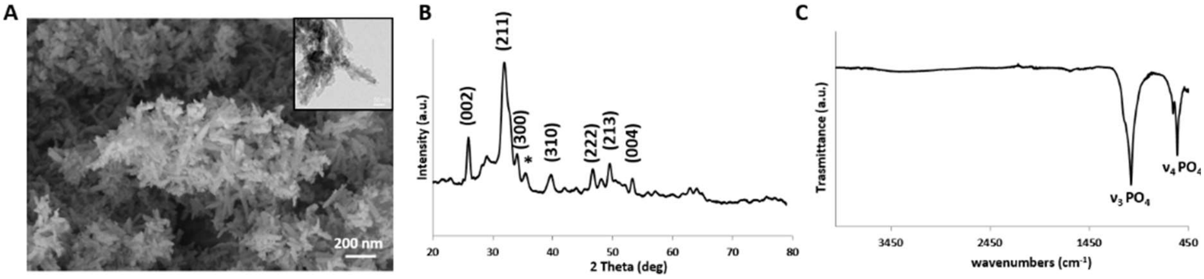

Figure S3: Fe-HA NM characterization via A) SEM-FEG image, B) XRD spectrum, and C) FTIR spectrum. *relative to a secondary phase corresponding to maghemite nanoparticles.

The diffraction peaks are broad and poorly defined, indicating the presence of calcium phosphate nanocrystals (about 20 nm) with reduced crystal domains and crystalline order (Fig. S3B). $Fe^{2+/3+}$ ions replace calcium in the lattice as in the disordered external layer. The XRD pattern also shows the occurrence of a secondary phase of maghemite, quantified by Rietveld refinement < 5wt%. The hydrodynamic diameter of CaP-HA [1 mg/mL] is 163.70 nm, and the zeta-potential is -19.30 mV. The particles showed a chemical composition of: Ca 23±2 wt%, P 15±1 wt%, Fe 10±1 wt%, (Ca+Fe)/P molar ratio= 1.64 ± 0.04 (obtained through ICP-OES).

Fe-HA NM displayed the typical FTIR apatitic pattern characterized of a main broad band at 1030 cm^{-1} due to antisymmetric stretching mode of the apatitic PO_4 groups (ν_3PO_4). Other features emerge at 603 and 565 cm^{-1} (triply degenerated bending mode of the same groups, (ν_4PO_4) (Fig. S3C).

Ti-HA were characterized by DLS, zeta-potential, TEM, XRD and FTIR.

Ti-HA NM were synthetized following the protocol reported by *Adamiano et al.* [3] appearing as a white poorly crystalline powdered solid characterized by rod-like nanoparticles of calcium phosphate (long axis 75-100 nm, short axis 15-20 nm), agglomerated into micron-size clusters (Fig. S4A).

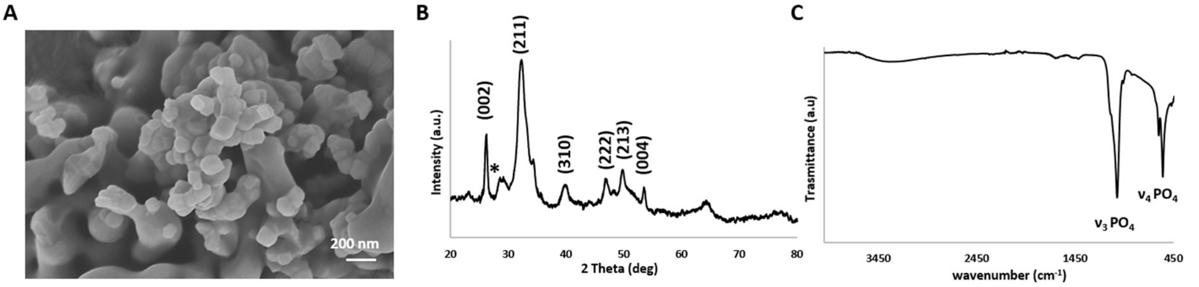

Figure S4: Ti-HA NM characterization via A) FEG-SEM image, B) XRD spectrum, and C) FTIR spectrum. *relative to a secondary phase corresponding to TiO_2 nanoparticles.

The diffraction peaks are broad and poorly defined, indicating the presence of calcium phosphate nanocrystals (about 20 nm) with reduced crystal domains and crystalline order (Fig S4B). No secondary crystalline phases are identified (e.g. TiO_2), highlighting as Ti ions are present inside the HA lattice. In particular, titanium ions (Ti^{4+} and TiO_4^{4-} ions) replace both calcium and phosphate ions in the lattice as in the disordered external layer as previously

reported [3]. The hydrodynamic diameter of Ti-HA [1 mg/mL] is 241.70 nm, and the zeta-potential is -9.11 mV. The particles showed a chemical composition of: Ca 37.0±1 wt%, P 14.6±1 wt%, Ti 4.5±1 wt%, (Ca+Ti)/(P+Ti) molar ratio = 1.49 ± 0.03 (obtained through ICP-OES).

Ti-HA NM displayed the typical FTIR apatitic pattern characterized of a main broad band at about 1030 cm−1 due to antisymmetric stretching mode of the apatitic PO4 groups (ν3PO4). Other features emerge at about 600 and 560 cm−1 (triply degenerated bending mode of the same groups, (ν_4PO_4) (Fig. S4C).

Ti-HA-Alg were characterized by SEM, TGA, XRD and FTIR.

Ti-HA-Alg NM were synthetized following the protocol patented by *Tampieri et al.* [4] appearing as a white to off-white pure crystalline powdered solid characterized by needle-like particles of 267 ± 76 nm length, with a high tendency to aggregate into hybrid flakes (Fig S5A and inset, respectively).

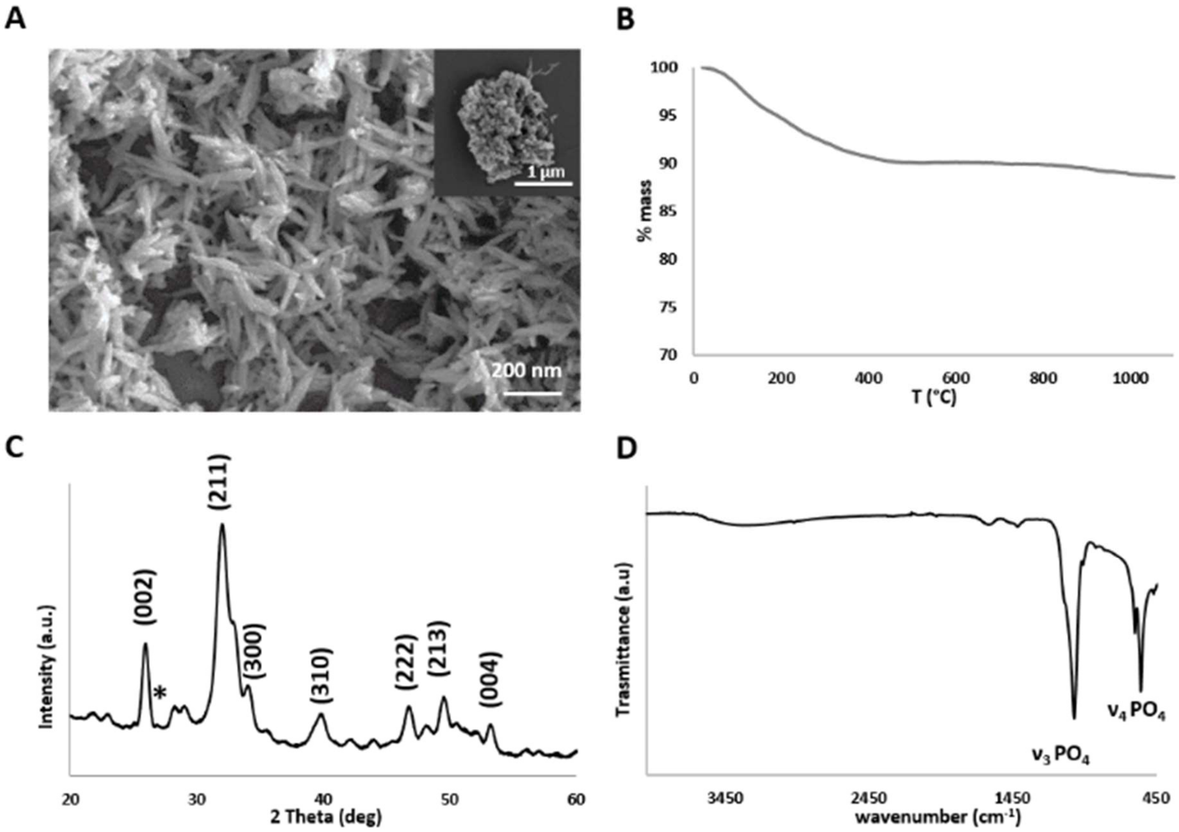

Figure S5: Ti-HA-Alg NM characterization via A) FEG-SEM image, B) TGA spectrum, C) XRD spectrum, and D) FTIR spectrum. *relative to a secondary phase corresponding to TiO_2 nanoparticles.

TGA confirmed that hybrid flakes are characterized by high mineral content; in particular Alg:TiHA ratio is lower than 20:80 (Fig. S5B). XRD profile showed the presence of a pure crystalline phase that can be identified as apatitic structure, however, XRD pattern is poorly resolved and with low intensity, typical of mineral phases with a low degree of crystallinity (Fig. S5C). No secondary crystalline phases are identified (e.g. TiO_2), highlighting as Ti ions are present inside the HA lattice. The hybrid particles showed a chemical composition of: Ti/Ca molar ratio= 15.0 ± 0.5%; Ti/P molar ratio= 23.2 ± 0.3%; (Ca+Ti)/(P+Ti) molar ratio= 1.49 ± 0.05 (obtained through ICP-OES).

Ti-HA NM displayed the typical FTIR apatitic pattern characterized of a main broad band at about 1030 cm−1 due to antisymmetric stretching mode of the apatitic PO4 groups (ν_3PO_4). Other features emerge at about 600 and 560 cm−1 (triply degenerated bending mode of the same groups, (ν_4PO_4) (Fig. S5D).

References

1. Landi, E.; Logroscino, G.; Proietti, L.; Tampieri, A.; Sandri, M.; Sprio, S. Biomimetic Mg-substituted hydroxyapatite: from synthesis to in vivo behaviour. *J. Mater. Sci. Mater. Med.* **2008**, *19*, 239–247, doi:10.1007/s10856-006-0032-y.

2. Tampieri, A.; D'Alessandro, T.; Sandri, M.; Sprio, S.; Landi, E.; Bertinetti, L.; Panseri, S.; Pepponi, G.; Goettlicher, J.; Bañobre-López, M.; et al. Intrinsic magnetism and hyperthermia in bioactive Fe-doped hydroxyapatite. *Acta Biomater.* **2012**, *8*, 843–851, doi:10.1016/j.actbio.2011.09.032.

3. Adamiano, A.; Sangiorgi, N.; Sprio, S.; Ruffini, A.; Sandri, M.; Sanson, A.; Gras, P.; Grossin, D.; Francès, C.; Chatzipanagis, K.; et al. Biomineralization of a titanium-modified hydroxyapatite semiconductor on conductive wool fibers. *J. Mater. Chem. B* **2017**, *5*, 7608–7621, doi:10.1039/C7TB00211D.

4. Tampieri, A.; Sandri, M.; Sprio, S. Physical solar filter consisting of substituted hydroxyapatite in an organic matrix 2022.
